# P04-08 The art of ageing well - a salutogenic study of physically active old adults

**DOI:** 10.1093/eurpub/ckac095.062

**Published:** 2022-08-29

**Authors:** Helena Ericson, Susanna Geidne, Mikael Quennerstedt

**Affiliations:** Örebro University, School of Health Sciences, Örebro, Sweden

**Keywords:** health resources, active ageing, salutogenic, ageing well, physical activity

## Abstract

**Background:**

People aged 60 years and over has doubled since 1980 and WHO predicts that this population will reach 2 billion by the year 2050. However, increases in life spans do not directly lead to increases in health. An aging population poses both challenges and opportunities for society and for individuals. In order to address this, scholars argue for the benefits of being physically active, especially in a group of peers. However, the relation between physical activity and health is often based on an understanding of what causes or prevents illness rather than what promotes health. The purpose of this study is thus to contribute to knowledge about which health resources older adults develop in their participation in organised physical activity initiatives. The study will consider to what extent older adults develop health resources, differences in demographic background and the relation between the health resources and Sense of coherence (SOC).

**Methods:**

This is the first data collection in a longitudinal study. Participants were old adult men and women, 60 years and above. All participants were active in ongoing organised physical activity initiatives in different organisations on a voluntary basis. A survey included demographics, overall health, health resources (McCuaig & Quennerstedt, 2018) and SOC-13. The data collection is ongoing (preliminary n = 200) and ends spring 2020. Statistical analyses were descriptive and included bivariate analyses.

**Results:**

Preliminary results show that the most frequent health resources are social relations, positive energy and embodied identity for both men and women. A positive related correlation of the health resource habit of exercising were observed with a high sense of coherence.

**Conclusion:**

The Salutogenic idea of having access to various health resources linked to a high sense of coherence is in line with the result of a positively related correlation direction and also with the health resource habit of exercising. The results of the study can contribute to knowledge about which health resources older adults develop in their participation in organised physical activity initiatives.

